# Adjunctive postoperative course of dexamethasone in chronic subdural hematoma: Effect on surgical outcome

**DOI:** 10.12669/pjms.37.7.3374

**Published:** 2021

**Authors:** Jibran Tariq, Sajid Nazir Bhatti

**Affiliations:** 1 Jibran Tariq, MS. Senior Registrar, Department of Surgery, Peshawar Institute of Medical Sciences, Pak International Medical College, Peshawar, Pakistan; 2 Sajid Nazir Bhatti, FCPS/MS. Head of Department, Department of Neurosurgery, Pakistan Institute of Medical Sciences, Shaheed Zulfiqar Ali Bhutto Medical University, Islamabad, Pakistan

**Keywords:** Chronic subdural hematoma, Recurrence, Dexamethasone

## Abstract

**Objectives:**

To compare the effect of burrhole craniostomy with and without a postoperative course of dexamethasone on recurrence rate of chronic subdural hematoma (CSDH).

**Methods:**

The study was conducted at the Department of Neurosurgery, Pakistan Institute of Medical Sciences, Islamabad, from September 2017 till May 2018. Adult patients diagnosed with CSDH and advised burrhole craniostomy were enrolled in this prospective randomized controlled trial. Participants were randomized into Group-1 (receiving two weeks dexamethasone), and Group-2 (no dexamethasone). Clinical assessment and Brain CT were done preoperatively, second postoperative day, sixth and twelfth postoperative week, with outcome assessed at twelfth postoperative week. Complications of treatment and recurrence rate were recorded.

**Results:**

Ninety-two (n=92, 46 in each group) patients were enrolled. Improvement in neurological (95.7% vs 93.5%; *P*=0.646) and radiological outcome (95.7% vs 93.5%; *P*=0.646) was similar in both groups. Complication rate was higher in Group-1 but not significantly different (58.7% vs 43.5%; *P*=0.535). Most frequent complication was pneumocephalus, with mortality rate equal (n=one). Recurrence was observed in 2.2% (n=1/46) patients in Group-1 and 4.3% (n=2/46) in Group-2 (*P*=0.557), which was not statistically significant.

**Conclusions:**

Neurological and radiological outcome, and mortality rates were similar in both groups. The recurrence rate was lower and complications slightly higher in Group-1 but these were not statistically significant.

## INTRODUCTION

Chronic subdural hematoma (CSDH) is a self-perpetuating inflammatory process of dura matter, wherein fluid collects within the dural border cell layer of the meninges. Brain atrophy causes stretching of subarachnoid and associated bridging veins making them susceptible to minor trauma, resulting in blood or cerebrospinal fluid leaking into the dural border cell layer causing local dissection.^1^ A membrane forms around this collection, wherein inflammatory cells are recruited and activated, becoming a source of angiogenic and inflammatory factors.^2^ A correlation exists between vascular-endothelial growth factor (VEGF) concentration within this fluid and exudation rate from membrane vessels.^3^ VEGF causes angiogenesis that undergoes recurrent micro-bleeding, resulting in increasing hematoma size. Compared to systemic levels, higher ratios of inflammatory to anti-inflammatory mediators exist within the hematoma,^4^ promoting angiogenesis, inflammation, hyper-permeability, and progression.

CSDH usually presents in the elderly, many with pre-existing history of mild head injury^5^ or comorbidities.^6^ Although surgery is treatment of choice, literature provides conflicting views on optimal management or surgical approach. Mortality doesn’t significantly differ between approaches and is attributed to comorbidities, with burrhole craniostomy having the best cure to complication ratio.^7^ Recurrence occurs in nine to twenty-two percent of surgically treated patients,^6^,^8^ causative factors being local inflammation, capillary leakage, angiogenesis, and membrane maturation.^9^ High levels of inflammatory mediators and angiogenic factors within the fluid collection are associated with recurrence.^3^,^4^ Corticosteroids may help by promoting anti-inflammatory pathways and vessel maturation, leading to hematoma resolution and reduced recurrence.^10^ Dexamethasone is an inflammatory inhibitor showing suppressive effects on VEGF production in rat models.^11^ Our study aimed to compare burrhole craniostomy with and without a two-week postoperative course of dexamethasone in terms of outcomes and recurrence rate of CSDH in our setting.

## METHODS

This study was conducted at Department of Neurosurgery, Pakistan Institute of Medical Sciences, Islamabad, Pakistan after approval from ethical and scientific board. Ethical Review Board approval reference number is F. 1-1/2015/ERB/SZABMU/, approved on 26^th^ September 2017. Study was conducted from September 2017 till May 2018. Total of ninety-two (n=92) patients were included (sample size calculated using WHO calculator ‘2.2b’ taking 5% significance level, 80% power of the test, anticipated population Proportion-I as 92%, anticipated population proportion-II as 77%).^12^ Informed consent was obtained from participants. Adult patients of either gender diagnosed with CSDH and advised burrhole craniostomy were enrolled. Exclusion criteria included pregnancy, decompensated liver/kidney disease, recent myocardial infarction, pre-existing infection or steroid therapy, and dexamethasone hypersensitivity. Subjects were randomized into two groups of forty-six (n=46) by lottery. Group-1 was administered sixteen mg dexamethasone preoperatively, followed by evacuation via surgery. Dexamethasone was then tapered over a total of fifteen days. Sixteen mg dexamethasone was administered in four divided doses per day for the first two postoperative days, and tapered in three mg decrements every three days. Group-2 underwent evacuation via surgery but did not receive dexamethasone. Surgery entailed single or double burrholes, followed by irrigation of hematoma space with room temperature normal saline (0.9% NaCl) and subdural drain placement.

Neurological status was assessed by GCS score, degree of hemiparesis, or presence of aphasia. It was considered improved if these parameters were better at last follow-up compared to at presentation, or deteriorated if worsening occurred at any time. Midline shift of brain was measured on Brain CT. Hematoma volume was calculated by measurements on Brain CT with formula [½x(a)x(b)x(c)], ‘a’ being maximum width, ‘b’ maximum length and ‘c’ number of consecutive 5 mm slices of Brain CT with hematoma. Radiological outcome was considered improved if decreased volume of hematoma was observed at last follow-up compared to the second postoperative day, and worsened if any increase occurred.

Both groups received postoperative antibiotics and analgesics for one week. Minimum postoperative hospital stay was three days, or longer depending on recovery. Follow-up visits with Brain CT at sixth and twelfth postoperative week were scheduled to ascertain neurological and radiological status. Outcomes were measured at twelfth postoperative week. Complications of treatment and recurrence were recorded. Data was analyzed using SPSS version 23. Paired sample t-test was used to compare changes in continuous variables from baseline at different time intervals. Chi-square test was used to compare categorical variables between both groups. *P*-value of ≤ 0.05 was considered significant.

## RESULTS

Age and gender distribution were similar in both groups (Table-I). Mean GCS, hematoma volume and midline shift showed significant improvement in both groups compared to baseline at day two, week six and week twelve (Table-II). Improvement rate in neurological and radiological outcomes was higher in Group-1 but not statistically significant (*P*=0.646 and 0.646 respectively, Table-III). Complication rate was higher in Group-1 but not statistically significant (58.7% vs 43.5%, *P*=0.535, Table-IV). Most frequent complication was pneumocephalus and one mortality occurred in both groups. Recurrence was observed in 2.2% (n=1/46) patients in Group-1 and 4.3% (n=2/46) in Group-2 (*P*=0.557).

**Table I:** Baseline information.

*Variables*	*Group-1 (dexamethasone)*	*Group-2 (control)*
(control)Age (Mean ± SD)	62.7±12.9	63.8±12.7
***Gender n (%)***		
Males	34 (73.9%)	33 (71.7%)
Females	12 (26.1%)	13 (28.3%)
***Comorbidities n (%)***		
None	23 (50.0%)	21 (45.7%)
Diabetes	5 (10.9%)	8 (17.4%)
Hypertension	5 (10.9%)	7 (15.2%)
Ischemic Heart Disease	0 (0%)	1 (2.2%)
Multiple	13 (28.2%)	9 (19.5%)
***Drug History n (%)***		
None	26 (56.5%)	21 (45.7%)
Antiplatelet	1 (2.2%)	2 (4.3%)
Oral Hypoglycemic	4 (8.7%)	6 (13.0%)
Antihypertensives	3 (6.5%)	6 (13.1%)
Insulin	0 (.0%)	1 (2.2%)
Multiple	12 (26.1%)	10 (21.7%)
***Neurological Status***		
Hemiparesis	42 (91.3%)	40 (87.0%)
Aphasia	2 (4.3%)	1 (2.2%)
None	2 (4.3%)	5 (10.9%)
***GCS***	
(Mean ± SD)	12.9±2.1	12.3±2.5
***Hematoma volume***		
(Mean ml ± SD)	136.6±75.4	175.1±101.1
***Midline shift on CT***		
(Mean mm ± SD)	16.5±7.5	17.3±7.6

**Table II:** Change in GCS, hematoma volume, and midline shift from baseline at different time intervals.

*Group-1 (dexamethasone)*	*Baseline*	*Day 2*	*Week 6*	*Week 12*
***GCS***				
(Mean ± SD)	12.9±2.1	14.6±1.01	15.0±0.001	14.8±2.2
P-Value (Paired t-test)	(0.001 at all time intervals)			
***Hematoma Volume***				
(Mean ml ± SD)	136.6±75.4	43.1±96.7	4.1±13.7	0.11±0.74
P-Value (Paired t-test)	(0.001 at all time intervals)			
***Midline Shift***				
(Mean mm ± SD)	16.5±7.5	2.9±4.1	0.33±1.6	0.001±0.0001
*P-Value* (Paired t-test)	(0.001 at all time intervals)			
***Group-2 (control)***				
GCS				
(Mean ± SD)	12.3±2.5	14.5±1.2	14.5±1.7	14.8±1.3
P-Value (Paired t-test)	(0.001 at all time intervals)			
***Hematoma Volume***				
(Mean ml± SD)	175.1±101.1	34.5±24.3	2.8±8.4	3.9±18.6
P-Value (Paired t-test)	(0.001 at all time intervals)			
***Midline Shift***				
(Mean mm± SD)	17.3±7.6	3.1±3.4	0.11±0.74	0.43±2.1
P-Value (Paired t-test)	(0.001 at all time intervals)			

**Table III:** Outcome at week 12.

*Variables*	*Group-1 (dexamethasone)*	*Group-2 (control)*	*p-value*
***Neurological n (%)***			
Improved	44 (95.7%)	43 (93.5%)	0.646
Deteriorated	2 (4.3%)	3 (6.5%)	
***Radiological n (%)***			
Improved	44 (95.7%)	43 (93.5%)	0.646
Deteriorated	2 (4.3%)	3 (6.5%)	

**Table IV:** Complications and recurrence rate in study groups.

*Variables*	*Group-1 (dexamethasone)*	*Group-2 (control)*	*p-value*
Complications n (%)	27 (58.7%)	20 (43.5%)	0.535
Pneumocephalus	17 (37.0%)	16 (34.8%)	
Decreased Diabetic Control	2 (4.3%)	0 (0%)	
Surgical Site Infection	1 (2.2%)	0 (0%)	
Iatrogenic Parenchymal Injury	3 (6.5%)	2 (4.3%)	
Mortality	1 (2.2%)	1 (2.2%)	
Multiple	3 (6.5%)	1 (2.2%)	
***Recurrence n (%)***			
Present	1 (2.2%)	2 (4.3%)	0.557
Absent	45 (97.8%)	44 (95.7%)	

Patients that developed complications were administered treatment accordingly. Recurrence required repeat surgery. Superficial surgical site infections were treated with culture-sensitive antibiotics and pneumocephalus with supplemental oxygen. Decreased diabetic control was managed with intensified insulin regimens, and referred for medical consultation after discharge. Patients with subdural empyema were administered intravenous antibiotics and operated for empyema evacuation.

Two deaths occurred during this study period, one in each group. One was a 65 years old lady with diabetes and hypertension presenting with hemiparesis and randomized into Group-2. Her hemiparesis improved postoperatively and was discharged in satisfactory condition on third postoperative day. She presented again three weeks later with fever, decreased conscious level, and worsening hemiparesis. Brain CT revealed subdural collection. She was re-operated via previous burrholes and confirmed to have subdural empyema. Subdural drain was placed and samples sent for culture-sensitivity, and appropriate antibiotics were initiated. No improvement occurred postoperatively. She developed a severe lower respiratory infection and died due to respiratory failure and sepsis in the sixth postoperative week. The second death was a 70 years old gentleman presenting with decreased conscious level and hemiparesis with no comorbidities and randomized into Group-1. He did not improve postoperatively, and second day Brain CT showed pneumocephalus which was treated with supplemental oxygen. Repeat Brain CT after one week revealed a subdural collection. He was operated on via small craniotomy, wherein diagnosis of subdural empyema was confirmed, and was evacuated. Subdural drain was placed and samples sent for culture-sensitivity, and appropriate antibiotics were administered. No improvement occurred postoperatively. He developed severe lower respiratory infection, and died in third postoperative week due to respiratory failure and sepsis.

## DISCUSSION

Several international studies on corticosteroids in CSDH have been published. Steroid treatment in CSDH has been proposed in patients with comorbidities.^12^ The risk of serious adverse events is low when using high-dose corticosteroids in the short term.^13^ Qian et al,^14^ suggest dexamethasone should be administered in addition to surgery when risk factors for recurrence are present, e.g. advanced age or midline displacement. Berghauser et al.^15^ retrospectively analyzed four-hundred and ninety-six patients with CSDHs. Mean age (71.5 ± 13.3 years) and male-to-female ratio (3:1) of study subjects were similar to our study. They demonstrated preoperative corticosteroids resulted in lower recurrence rate and mortality risk in CSDH treated with burrhole craniostomy, with no increase in complication rates. We found similar trends in our study with recurrence being lower in patients where postoperative dexamethasone was used, whereas complication rate was higher, none of which were statistically significant. Dran G et al.^16^ enrolled one-hundred and forty-two patients treated with methylprednisolone and surgery, and fifty-six underwent only surgery. They showed corticosteroids significantly reduced mortality. We did not see any difference in our mortality rates. Patients in their study received four weeks of corticosteroids, whereas ours received two weeks. Delgado-López et al.^17^ reported better outcomes in patients treated with adjunctive dexamethasone compared to only surgery. Sun, et al.^12^ randomized one-hundred and twelve patients into four groups: burrhole irrigation, dexamethasone with surgery, dexamethasone without surgery, and conservative medical treatment. They started dexamethasone forty-eight hours preoperatively and tapered over two weeks. Their reoperation and mortality rates were not statistically significant in any group (P>0.05). These results are similar to ours, with no statistically significant difference between our groups.

Various studies suggest corticosteroids help in the treatment of CSDH and lower recurrence rates.^15^,^18^ A major randomized controlled trial is underway studying the effects of dexamethasone therapy on the outcome of chronic subdural hematoma.^19^ A recent large multi-center trial (Dex-CSDH) showed that adjuvant dexamethasone resulted in reduced need for repeat operations, but was associated with adverse events, and slightly less favorable outcomes compared to not using dexamethasone.^20^ In contrast, another study showed that patients have better outcomes when treated with surgery combined with corticosteroids.^21^ Interim analysis of yet another trial suggests adjuvant dexamethasone can safely reduce recurrence.^22^

In our study, patients receiving dexamethasone had lower recurrence rates and more adverse effects (Table-IV), both of which were not statistically significant. No statistical significance was found in difference of neurological/radiological outcomes, or mortality.

To our knowledge, this is the first study conducted in Pakistan concerning adjunctive dexamethasone treatment in CSDH. Dexamethasone is an easily available inexpensive drug, and safe when used in the short-term.^13^,^22^ Although not achieving statistically significance, our study suggests the use of dexamethasone in the treatment of CSDH results in lower recurrence rates, preventing the need for reoperation for this common condition. Lower recurrence rates have implications in reducing readmission, patient morbidity, and socio-economic impact of this disease on patient families and hospitals.

### Limitations

Our sample size was relatively small, yet sufficient to draw an inference. The follow-up period was short, being only twelve weeks, therefore could not assess long-term outcomes. This study was single-blinded and investigators aware of patient allocation. Different surgeons were involved in treatment, but all had similar clinical experience. A larger double-blinded study with longer follow-up duration might provide further information on recurrence rates and patient outcomes in both groups, and better able to highlight differences in statistically significant terms.

### Recommendations

We recommend a further prospective randomized controlled trial with a larger sample size and longer duration of follow-up to better determine the effectiveness of dexamethasone on recurrence rates and patient outcomes.

## CONCLUSION

Neurological and radiological outcome, and mortality rates were similar in both groups. The recurrence rate was lower and the complication rate higher in the dexamethasone group but both were not statistically significant.

### Authors’ Contribution:

**JT** conceived, designed study, collected data, statistical analysis, manuscript writing, editing, responsible/accountable for accuracy/integrity of study.

**SNB** supervision, critical review, editing and manuscript approval, responsible/accountable for accuracy/integrity of study.
